# Prevalence and outcomes of central venous catheter-related bacteraemia in HIV-infected versus non-HIV-infected patients undergoing haemodialysis treatment for end-stage kidney disease

**DOI:** 10.4102/sajhivmed.v19i1.859

**Published:** 2018-11-22

**Authors:** Nuria Avila-Danguillecourt, Anand A. Moodley, Polycarpe Makinga

**Affiliations:** 1 Department of Internal Medicine, Grey’s Hospital, University of KwaZulu-Natal, South Africa; 2 Department of Medicine, University of the Free State, South Africa; 3 Department of Family Medicine, Ladysmith Hospital, University of KwaZulu-Natal, South Africa

## Abstract

**Background:**

Central venous catheter (CVC) haemodialysis (HD) to implement renal replacement therapy is the preferred choice in the urgent setting. Unfortunately, CVC placement is associated with multiple complications including nosocomial bloodstream infections. There is a paucity of data on the prevalence and pattern of pathogenic organisms in haemodialysed HIV-infected versus non-HIV-infected patients with end-stage kidney disease.

**Method and results:**

We undertook a retrospective study of 228 patients who were dialysed using a CVC at a tertiary referral hospital in KwaZulu-Natal, South Africa. Seventy-eight patients (34.2%) complicated with bacteraemia and sepsis requiring antibiotics. Removal of the catheter was necessary in 58 patients (74.0%). The most common organisms isolated were *Staphylococcus aureus* (30.8%), *Staphylococcus epidermidis* (24.4%) and *Klebsiella pneumoniae* (15.4%). There was no statistically significant difference between HIV-infected and non-infected patients with regards to infection rate, time interval from insertion of CVC to infection and final outcome. However, HIV-infected patients took longer to recover; 54.3% of non-infected patients versus 10.3% HIV-infected patients had their sepsis controlled within one week. Acidosis, hypotension, line malfunction and line discharge were infrequent signs of sepsis. Fever, rigors and raised white cell count occurred in over 80.0% of patients.

**Conclusion:**

The infection rate in CVC HD is not more frequent in HIV-infected patients, provided that CD4+ count is ≥ 200 cells/µL and the patient is virologically suppressed. Outcomes following intravenous antibiotic and removal of the CVC are similar in HIV-infected and non-infected patients but response to treatment is slower in HIV-infected patients. A high index of suspicion is needed in detecting CVC-related bacteraemia.

## Introduction

The gold standard in management of chronic kidney disease (CKD) patients varies according to the stage of the disease. In early stages, the goal of management is to correct reversible insults and retard progression. However, in the late stages of the disease, renal replacement therapy (RRT) is needed to sustain and maintain life. In the South African setting this approach is not always possible owing to the late presentation of patients for medical care. Hence, there is often a need for urgent and temporary vascular access.

Central venous catheter (CVC) haemodialysis (HD) to implement RRT is the preferred choice in the urgent setting. Unfortunately, CVC is associated with multiple complications including nosocomial bloodstream infections. The infection rate of CVC is reported as high as 32.0% and mortality rate amongst the infected patients as much as 12.0% – 26.0%.^1^ The Kidney Disease Outcomes Quality Initiative (KDOQI) has recommended the use of the arteriovenous fistula (AVF) as the modality of choice for vascular access in end-stage kidney disease (ESKD).^2^ This is not always possible in resource-constrained settings as well as in those situations where the quality of the vasculature and time constraints prevent AVF formation. In such cases, long-dwelling intravenous catheters are used and remain a reliable option for HD despite the risk for infection. The three veins commonly used for CVC insertion are the femoral, internal jugular and subclavian veins.

Since its early days, CVC has undergone numerous advances in its design, insertion technique and ongoing care. However, the risk of infective complications remains higher in patients with CVC than in those with AVF.^3^ A meta-analysis published in 2006 by Pronovost et al. reported an incidence range of infection of between 1 and 3.1 per 1000 patients per day.^4^

South Africa has the largest human immunodeficiency virus (HIV) epidemic in the world. In 2015 it was estimated that seven million people were living with HIV in South Africa.^5^ At the same time South Africa has the largest antiretroviral treatment programme in the world. Despite having the largest antiretroviral programme, the prevalence of HIV remains high, the highest being in KwaZulu-Natal (40.0%) and the lowest in Northern and Western Cape (18.0%).^6,7^ The HIV epidemic continues to burden the delivery of optimal healthcare in South Africa. Despite the availability of antiretroviral therapy (ART), HIV-associated nephropathy (HIVAN) is still highly prevalent because of the virus itself and ART, specifically tenofovir.^8^ HIVAN is characterised histologically by a collapsing form of focal segmental glomerulosclerosis, microcystic tubular dilation, interstitial inflammation and fibrosis.^8^ It is a result of the direct effect of HIV-1 and the expression of viral genes on the renal epithelial cells in a genetically susceptible host.^9^ Other reported glomerular lesions in HIV-infected patients include cryoglobulinaemia, IgA nephropathy, amyloidosis and a lupus-like immune complex glomerulopathy.^10^ In the pre-ART era, HIVAN was characterised by rapid progression to kidney failure and ESKD, leading to the need for dialysis.

HIV infection is not a contraindication for inclusion into the chronic renal programme in South Africa as long as the patient’s CD4+ count is ≥ 200 cells/µL and the patient is virologically suppressed.^1^ This encouraging development has brought new challenges to the management of HIV-infected patients with ESKD. Conceivably, the risk of infection in haemodialysed HIV-infected patients even with mild immunosuppression should be higher than the non-immunocompromised population. When line sepsis is suspected, patients are started empirically on antibiotics until culture and antibiotic sensitivity results are obtained.

There is a paucity of data on the prevalence and pattern of pathogenic organisms in the haemodialysed HIV-infected group, specifically with relation to CVC-related infections. There is no study conducted in South Africa to determine whether the rate of central venous catheter-related bacteraemia (CVC-RB) in HIV-infected patients is comparable to that of non-infected patients. The spectrum of pathogens implicated in CVC-RB varies across different data sources. According to data from the USA, the common pathogens causing CVC-RB are coagulase negative *Staphylococcus* (32.0% – 45.0%), *Staphylococcus aureus* (22.0% – 29.0%) and *Enterococcus* (9.0% – 13.0%).^11^ In a South African study by Bisiwe et al., *Staphylococcus* spp. were the most common pathogens isolated, but no comparison was made between HIV-infected and non-infected individuals.^12^

We undertook a retrospective chart review to address this by investigating the prevalence of CVC-RB in HIV-infected and non-infected patients with ESKD at Grey’s Hospital, a tertiary hospital based in KwaZulu-Natal, South Africa. Our aim was to determine the commonest microbial agents implicated in CVC-RB, in HIV-infected versus non-HIV-infected subjects, and to compare the outcome of the current treatment protocols in HIV-infected versus non-infected subjects.

Our study population included patients with ESKD treated with HD via a CVC and complicated by CVC-RB.

## Method

A retrospective cross-sectional chart review was conducted in the renal unit of Grey’s Hospital, a 494-bed tertiary hospital in Pietermaritzburg, South Africa. The hospital serves an urban population of 860 000 people and a rural population of approximately four million. Patients diagnosed with ESKD are referred to Grey’s Hospital for RRT from its referring hospitals. The majority of these cases present late in the course of the disease and often require urgent dialysis. The CVC placement is done as an emergency procedure in many cases. On average 80–90 patients are dialysed monthly with about 600 sessions of HD per month. The study population in this case comprised all patients with ESKD attending Grey’s Hospital. All patients with ESKD receiving HD treatment via CVC at Grey’s Hospital between 01 January 2013 and 31 December 2015 were eligible for inclusion in the study. Participants had to be 12 years of age or older and only cases with a first episode of infection related to CVC were considered.

All patients with suspected line sepsis are treated empirically with antibiotics. The renal unit protocol at Grey’s Hospital is vancomycin 1 g stat intravenously and, depending on the stability of the patient, additional antibiotics are given. For stable patients, intravenous coamoxiclavulanate 1.2 g eight-hourly and for unstable patients intravenous meropenem 1 g eight-hourly with or without intravenous gentamicin 240 mg daily is given. Despite the renal failure, infection control takes precedence as it is lifesaving. Infection control is defined as response to treatment by clinical and laboratory markers. If the patient’s temperature, pulse rate, blood pressure, white cell count and C-reactive protein normalise, the infection is regarded as controlled. If after seven days of intravenous antibiotics, there is persistence of fever, hypotension, tachycardia and neutrophilia, antibiotics are continued. When culture and sensitivity results are available, appropriate changes to treatment are made. A delayed response is considered if infection control occurs beyond 10 days of antibiotic usage. The catheters are removed if the infection does not subside, there is poor quality dialysis or the catheter is non-functioning as a result of the infection. Furthermore, if there is abscess formation at the insertion site, then removal of the catheter promotes infection control. This protocol is followed equally for HIV-infected and non-HIV-infected patients.

Patients with acute renal failure, those whose source of infection was found not to be CVC-related and those whose HD was delivered through AVF were excluded from analysis. Only patients with long-dwelling CVC were included in the analysis.

Several data sources were consulted for both the sample selection and the actual data collection. These included the renal unit patient database, the infectious disease patient database, the National Health Laboratory Services database and patients’ medical records. Data were collected using a predesigned structured data collection sheet.

After collection, data collection sheets were checked for accuracy and completeness and were entered into a computer database by a single data-capturer, and where discrepancies were found data cleaning was performed.

**Statistics:** Data were analysed using IBM^®^ SPSS^®^ (version 21) software. Descriptive statistics in the form of frequency tables, proportions and summary statistics for continuous variables were computed. The chi-square tests of association and Student’s *t*-test were computed to assess associations and compare means at the significance level of 5.0%. The Fischer exact test was also computed for comparative analysis with a significance level of 5.0%.

## Ethical consideration

Ethical clearance was obtained from the ethics committee of the University of KwaZulu-Natal. Given that this was a retrospective chart review using records of patients seen at the hospital two – three years ago, the consent to use these records was obtained from the hospital management and not from individual patients. Information collected from these records was kept confidential.

## Results

**FIGURE 1:**
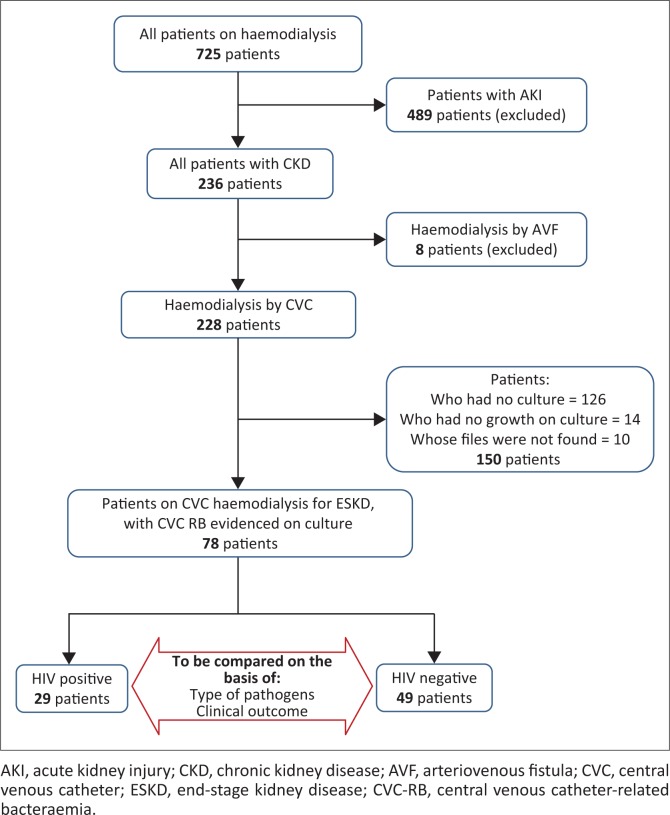
Patient selection.

There were 725 patients admitted for renal impairment in Grey’s Hospital during the two-year study period. Of these, 236 patients (32.6%) were diagnosed as having CKD. Of the 236 patients with CKD, 228 patients (96.6%) had HD through a CVC. Of the 228 who had CVC, microscopy, culture and sensitivity were performed on 92 (40.4%) patients, from which 78 (84.8%) patients were culture infected and had organisms identified. The remaining 14 (15.2%) had no growth (Figure 1).

These 78 patients with CVC-RB were further analysed. Sixty-two (79.5%) patients had internal jugular catheters, 10 (12.8%) patients had subclavian catheters and six (7.7%) patients had femoral catheters. In the group of patients with CVC-RB, 29/78 (37.0%) were HIV-infected and 49/78 (63.0%) were not HIV-infected. There was no significant difference in the CVC insertion site between the HIV-infected and non-infected patients. The sample was predominately male (56.4%). The mean age of participants was 38.1 years (s.d. = 14.6) with a range from 13 to 78 years. About 35.9% of patients were employed while only 5.1% were on a government-funded pension. The rest of the participants were either scholars or unemployed.

All 29 (37%) HIV-infected patients were on first-line ART and were virologically suppressed. First-line ART in the South African public health sector includes tenofovir, lamivudine (or emtricitabine) and efavirenz.^6^ Viral suppression was defined as viral load undetectable or < 50 copies/mL. All patients had a CD4+ count ≥ 200 cells/µL. Fifteen patients had a CD4+ count of > 500 cells/µL.^6^

The commonest comorbidities were anaemia of chronic diseases (68 patients), secondary hypertension (22 patients) and hypercholesterolaemia (six patients). There was no significant difference in the comorbidities between the HIV-infected and non-infected groups. Table 1 shows the aetiology of ESKD in the two groups. The cause of ESKD was unknown in eight patients; related to HIV infection alone in 12/29 (41.0%) patients; diagnosed on biopsy in five patients; and on ultrasound and clinical grounds in seven patients. Ten (34.0%) HIV-infected and 12 (24.0%) non-infected patients had multiple causes of ESKD as detailed in Table 1.

**TABLE 1:** Aetiology of end-stage kidney disease.

Aetiology	HIV-infected	Non-HIV-infected	Total		*p* (Fischer’s exact test)
			*n*	%	
**Single cause**					
HIV	12	0	12	5.4	-
Hypertension	0	7	7	8.0	-
Autoimmune diseases	1	7	8	10.3	0.07
Nephrotic syndrome	1	7	8	10.3	0.07
NSAIDs	3	2	5	6.4	1.00
Renal artery stenosis	1	1	2	2.6	1.00
Diabetes	0	2	2	2.6	-
Ureterocoele	1	0	1	1.3	-
Cervical cancer	0	1	1	1.3	-
Neurogenic hypoplastic bladder	0	1	1	1.3	-
Chronic glomerulonephritis	0	1	1	1.3	-
**Multiple causes**					
Hypertension and diabetes	2	7	9	11.5	0.18
HIV and NSAIDs	5	0	5	6.4	-
Hypertension and nephrotic syndrome	0	2	2	2.6	-
HIV and diabetes	2	0	2	2.6	-
Hypertension and NSAIDs	0	1	1	1.3	-
Urate nephropathy and NSAIDs	0	1	1	1.3	-
Diabetes and nephrotic syndrome	0	1	1	1.3	-
Hypertension and diabetes and HIV	1	0	1	1.3	-
**Unknown causes**					
Unknown	0	8	8	10.3	-
**Total**	**29**	**49**	**78**	**100.0**	**-**

NSAIDs, non-steroidal anti-inflammatory drugs.

Note: Dashes indicate *p*-value cannot be computed for these particular aetiologies because one of the cells contains zero observations.

Before blood culture was performed to confirm the diagnosis of bacteraemia, infection was suspected on the basis of clinical and laboratory markers. The clinical and laboratory markers present in more than 75.0% of patients were as follows: raised white cell count in 78 patients (100.0%), raised neutrophils in 76 patients (97.4%), rigors in 72 patients (92.3%), fever in 65 patients (83.3%) and tachycardia in 59 patients (75.6%) (Table 2). In HIV-infected patients, the erythrocyte sedimentation rate is commonly elevated and therefore not considered a useful marker of sepsis in this group.

**TABLE 2:** Clinical and laboratory findings suggestive of sepsis.

Signs	Present		Absent		Not done		p* (Fischer’s exact test)
	*n*	%	*n*	%	*n*	%	
**Clinical signs**							
Fever	65	83.3	12	15.4	1	1.3	0.00
Rigors	72	92.3	6	7.7	0	0.0	0.00
Tachycardia	59	75.6	19	24.4	0	0.0	0.00
Hypotension	23	29.5	51	65.4	4	5.1	0.00
Line malfunction	30	38.5	45	57.7	3	3.8	0.11
Line discharge	14	17.9	60	76.9	4	5.1	0.00
**Laboratory signs**							
Raised WCC	78	100.0	0	0.0	0	0.0	-
Raised neutrophils	76	97.4	1	1.3	1	1.3	0.00
Raised CRP	55	70.5	23	29.5	0	0.0	0.00
Acidosis	12	15.4	46	59.0	20	25.6	0.00

WCC, white cell count; CRP, C-reactive protein.

Note: Dash ‘-’ indicates *p*-value could not be computed for this particular WCC because the cell in the ‘Absent’ column contains zero observations.

* The proportions contained in the ‘Not done’ column were not included in the computation of the *p*-value. Only the proportions in the ‘Present’ and ‘Absent’ columns were compared.

**FIGURE 2:**
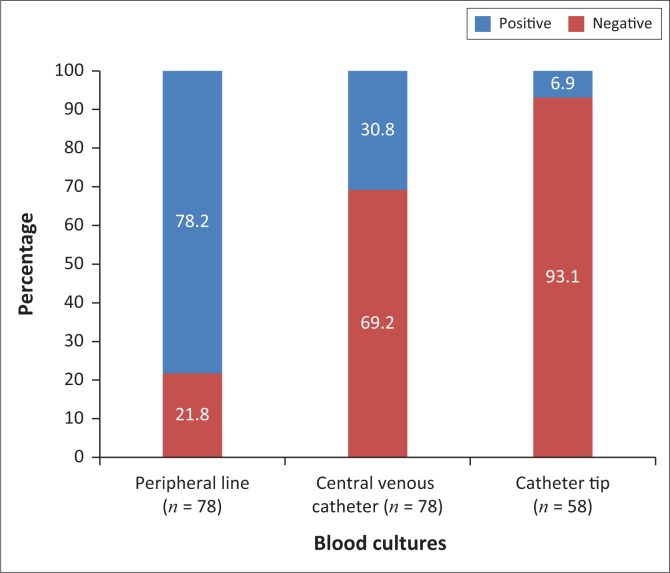
Percentage of positive blood cultures from different sites in 78 cases. Fifty-eight patients had the catheter removed.

Three specimens were collected from each patient: one from the peripheral line, one from the CVC and the third from the tip of the catheter when the catheter was removed. The proportions of patients who had an infected growth on specimens from one, two and three sites were 50.0%, 44.9% and 5.1%, respectively. Of the 78 cases with CVC-RB, blood from the peripheral line yielded infected growth in 17/78 (21.8%), from the CVC in 54/78 (69.2%) and from the tips of the catheter in 54/58 (93.1%). In 58 cases the catheter was removed and a specimen from the tip was cultured (Figure 2).

The incidence rate of bacteraemia events was 2.7 per 1000 catheter-days for HIV-infected individuals, 3.7 per 1000 catheter-days for non-infected individuals and 3.2 per 1000 catheter-days for the entire sample of 78 patients, as shown in Table 3. The frequently isolated pathogens were *Staphylococcus aureus* in 24 patients (30.8%), *Staphylococcus epidermis* in 19 patients (24.4%), *Klebsiella pneumoniae* in 12 patients (15.4%), *Staphylococcus haemolyticus* in eight patients (10.3%), *Escherichia coli* in four patients (5.1%) and *Pseudomonas aeruginosa* in four patients (5.1%) (Table 4). Where the Fischer exact tests could be computed, no statistical difference in the occurrence of these organisms between the HIV-infected and non-infected groups could be found.

**TABLE 3:** Incidence of bacteraemia.

Variable	HIV-infected	Non-HIV-infected	Total
Number of patients	29	49	78
Number of bacteraemia events	29	49	78
Catheter-days (total sum of numbers of days prior to infection onset for all patients)	10 838	13 339	24 177
Incidence rate (number of bacteraemia events per 1000 catheter-days)	$\frac{29}{10\;838}\times 1000=2.7$ 2.7 bacteraemia events per 1000 catheter-days	$\frac{49}{13\;339}\times 1000=3.7$ 3.7 bacteraemia events per 1000 catheter-days	$\frac{78}{24\;177}\times 1000=3.2$ 3.2 bacteraemia events per 1000 catheter-days

**TABLE 4:** The prevalence and antibiotic sensitivity of isolated microorganisms.

Variable	HIV-infected	Non-HIV-infected	Total		*p* (Fischer’s exact test)
			*n*	%	
**Gram-positive bacilli**					
*Staphylococcus aureus*	9	15	24	30.8	0.31
*Staphylococcus epidermis*	7	12	19	24.4	0.36
*Staphylococcus haemolyticus*	2	6	8	10.3	0.29
*Enterococcus faecalis*	2	1	3	3.8	1.00
*Corynebacterium species*	0	3	3	3.8	-
*Staphylococcus warneri*	2	0	2	2.6	-
*Bacillus species*	1	0	1	1.3	-
*Corynebacterium diphtheriae*	1	0	1	1.3	-
Non-fermenting gram-positive bacillus	0	1	1	1.3	-
*Staphylococcus* (coagulase negative)	0	1	1	1.3	-
*Staphylococcus dysgalactiae*	0	1	1	1.3	-
*Staphylococcus hominis*	1	0	1	1.3	-
*Staphylococcus lentus*	1	0	1	1.3	-
*Streptococcus pyogenes*	1	0	1	1.3	-
**Gram-negative bacilli**					
*Klebsiella pneumonia*	2	6	12	15.4	0.29
*Pseudomonas aeruginosa*	2	2	4	5.1	1.00
*Escherichia coli*	1	3	4	5.1	0.63
*Proteus mirabilis*	0	3	3	3.8	-
*Morgamella morganii*	1	2	3	3.8	1.00
*Acinetobacter baumania*	0	2	2	2.6	-
*Candida albicans*	1	0	1	1.3	-
*Enterobacter amnigenus*	1	0	1	1.3	-
*Stenotrophomonas maltophilia*	0	1	1	1.3	-

Note: Dashes indicate *p*-values could not be computed for these particular organisms because the cell in one of the columns contains zero observations.

The mean lag time between the insertion of the catheter and the onset of bacteraemia was 373.7 days (s.d. 251.6) for the HIV-infected group and 272.2 days (s.d. 262.5) for the non-infected group. This too was not significantly different (*p* = 0.99).

The management of patients with CVC-RB required that a decision be made to remove or preserve the catheter, depending on the clinical circumstances. After such a decision was made, the patient was then put on single or combination antibiotics, given orally or intravenously. The majority of patients were treated by removing the catheter and commencing intravenous antibiotics. Fifty-eight (74.4%) patients had the CVC removed. Eleven patients (14.1%) were treated with triple therapy antibiotics while 48 (61.5%) and 19 (24.4%) were on dual therapy and monotherapy, respectively. In this unit, vancomycin was the commonest antibiotic used, both as single agent and in combination with other antibiotics.

Of the 75 patients who had their infections controlled (determined on the basis of both laboratory and clinical grounds), 28 patients (37.3%) had their infection controlled within the first week of treatment while 41.3% and 21.3% needed two and more than two weeks, respectively. The stratification according to the HIV status presented in Table 5 shows that HIV-infected patients required more time to control infection than non-infected patients. For instance, 54.3% of non-HIV-infected patients versus 10.3% of HIV-infected patients had their infection controlled within one week. Only 15.2% of HIV non-infected patients versus 31.0% of HIV-infected patients required more than two weeks to control their infection. The Pearson chi-square test showed a statistically significant association between the patient’s HIV status and the time required to control infection: (*χ*^2^ = 14.73, *p* = 0.00), implying that the control of infection in HIV-infected patients took longer than in non-infected patients.

**TABLE 5:** Treatment outcomes of bacterial infection: HIV-positive versus HIV-negative.

Infection outcome	Count and Percent	HIV-positive	HIV-negative	Total
Infection controlled without complications	Count	24	42	66
	Percent within outcome %	36.4	63.6	100.0
	Percent within HIV status %	82.8	85.7	84.6
	Percent of total %	30.8	53.8	84.6
Infection controlled with complications	Count	5	4	9
	Percent within outcome %	55.6	44.4	100.0
	Percent within HIV status %	17.2	8.2	11.5
	Percent of total %	6.4	5.1	11.5
Infection not controlled leading to death	Count %	0	3	3
	Percent within outcome %	0.0	100.0	100.0
	Percent within HIV status %	0.0	6.1	3.8
	Percent of total	0.0	3.8	3.8
Total	Count	29	49	78
	Percent within outcome %	37.2	62.8	100.0
	Percent within HIV status %	100.0	100.0	100.0
	Percent of total %	37.2	62.8	100.0

## Discussion

Catheter-related bacteraemia is a serious complication that can lead to adverse clinical outcomes for the patient on HD. The prevalence rate of bacteraemia in HD patients with CVC was 34.1% at Grey’s Hospital, Pietermaritzburg, a 494-bed tertiary hospital serving a population of just under 5 million. This rate is more or less similar to the 32.0% found by Bisiwe et al. at Universitas Academic Hospital, Bloemfontein.^12^ Only patients who exhibit some clinical signs of infection warrant blood culture to exclude infection. A low threshold in ordering blood culture is paramount to diagnosing sepsis early.

Anaemia of chronic disease, hypertension (primary and secondary) and hypercholesterolaemia emerged as common comorbidities in 66 (87.2%), 30 (38.5%) and six (7.7%) patients, respectively. This result is in keeping with findings of other studies conducted in South Africa.^12^ Anaemia is a known complication of ESKD. Renal impairment leads to reduced production of erythropoietin by the failing kidney. Other causes of anaemia included blood loss (in cases of high incidence of bleeding, uraemic gastritis and HD) and reduced iron storage (iron deficiency anaemia). The causes and consequences of anaemia were not investigated in this study. Further research is needed to investigate in detail this important comorbidity in haemodialysed HIV-infected patients.

The common causes of ESKD were HIVAN (12 patients; 15.4%), the combination of hypertension and diabetes (nine patients; 11.5%), nephrotic syndrome (eight patients; 10.3%), autoimmune diseases (eight patients; 10.3%), hypertension (seven patients; 8.0%), non-steroidal anti-inflammatory drugs (NSAIDs) (five patients; 6.4%), NSAIDs and HIVAN (five patients; 6.4%) and NSAIDs and hypertension (one patient; 1.3%) as seen in Table 1. The role of tenofovir and HIVAN in the aetiology of ESKD may be difficult to distinguish in HIV-infected patients. This too was not investigated in detail in this study but may be of interest for further study. Of the 12 cases that had nephrotoxic drug use as cause of ESKD in isolation or in combination with other causes, tenofovir was the offending drug in eight cases, NSAIDs in three cases and herbal medicines in one case.

Acidosis cannot be relied on when diagnosing or suspecting infection as it was only present in 12/46 (26.0%) of patients. Clinical and laboratory signs present in more than 50.0% of patients were raised white cell count, raised neutrophils, rigors, fever, tachycardia and raised C-reactive protein. Clinicians should rely more on these signs to diagnose or suspect sepsis instead of acidosis, hypotension, line discharge or line malfunction, which were present in less than 75.0% of patients. In certain circumstances, line malfunction is not because of infection. Other causes of line malfunction include mechanical obstruction, line kinks, misplaced sutures, catheter migration, drug precipitation, catheter cracks and patient’s position.^2,13^ These factors should be kept in mind and ruled out before ordering blood culture for line malfunction. Raad et al. suggest that local catheter site inflammation is associated with a sensitivity of ≤ 3% for infection or may exist in the absence of CVC-RB and therefore cannot be relied upon. Patients with systemic signs of infections such as fever and chills should have their blood drawn for blood culture.^14^

*Staphylococcus aureus* remains the commonest pathogen in both HIV-infected and non-infected patients. This is in keeping with findings of several studies.^11,12,15,16^ In this study, it was difficult to make meaningful comparisons of the predominance of specific bacteria between the HIV-infected and non-infected groups, owing to the wide variety of organisms detected and small numbers. Mokrzycki et al. found a statistically significant fivefold increased risk of HIV-infected patients having a gram-negative organism.^15^ *Klebsiella pneumoniae, E. coli* and *P. aeruginosa* were the most commonly isolated gram-negative bacilli in this study but not statistically more common in the HIV-infected group.

Surprisingly, the mean lag time between the time of catheter insertion and the onset of bacteraemia was slightly longer in the HIV-infected (373.7 days) than in the non-infected (272.2 days) patients. However, this difference was not statistically significant. The lack of difference in this lag time can be attributed to the fact that most (51.7%) HIV-positive patients enrolled in this study had a CD4+ count > 500 cells/µL, with the remainder having a CD4+ count of 200 cells/µL to 500 cells/µL, and their viral load was undetectable; hence there were fewer people profoundly immunosuppressed. The data do indicate that there was a statistically significant dependency between time needed to control sepsis and HIV status. The time required to control sepsis was longer in HIV-infected than in non-infected patients. Apart from the ongoing inflammatory process in HIV-infected patients we postulate that drug interactions between ART and antibiotics might also account for the delayed response.

In addition to antibiotic use, 58 patients (74.4%) had their catheter removed. According to the 2015 KDOQI clinical practice guidelines update, it is recommended that the catheter be replaced in most instances (guideline 7.4.3).^2^ In our setting, this has become a standard of care in the majority of cases; however, conservative management is used on patients with poor or previously damaged vasculature and good response to antibiotics.

Of the 12 patients who either died, developed complications or whose infection was not controlled, only five were HIV-infected, 11 had their catheter removed, two were on antibiotic monotherapy, five on dual therapy and five on triple therapy. There was no association between HIV status and the treatment outcome (*χ*^2^ = 5.27, *p* = 0.15). The infection was controlled in 82.8% (24/29) of HIV-infected patients versus 85.7% (42/49) of non-infected patients. The remaining 7.2% (5/29) of HIV-infected patients had complications or their sepsis was not controlled, while only 14.3% (7/49) of non-infected patients had complications or died. HIV infection does not impact negatively on outcome when the CD4+ count is ≥ 200 cells/µL and patients are virologically suppressed.

**Limitations:** The major limitations of the study were as follows: firstly, this was a retrospective study and some patients’ files were not obtainable. Secondly, only HIV-infected patients with a CD4+ count ≥ 200 cells/µL and who were virologically suppressed were included in the study, as this is a requirement for a patient to be admitted to the chronic renal programme. Severely immunocompromised patients are excluded from the chronic renal programme and therefore could not be part of the study sample. Thirdly, the number of patients from which specific microorganisms were isolated was too small to allow meaningful comparison between the HIV-infected and non-infected groups for a specific microorganism. Nevertheless, we do believe that the data presented are useful for making reliable estimates of the differences and similarities between HIV-infected and non-infected patients with ESKD, dialysed via CVC. Much to our surprise, HIV-infected patients complicated as much and recovered as much as non-infected patients.

## Conclusion and recommendations

Based on this study our conclusions and recommendations are as follows:

- The CVC-related bacteraemia at Grey’s Hospital renal unit is 34.1%, which is similar to findings at Universitas Hospital in the Free State. Such a high prevalence warrants regular check-up and screening for sepsis during follow-up of these patients.
- HIV status should not be used as the basis of exclusion of HIV-infected patients from RRT as long as these patients are virologically suppressed with a CD4+ count ≥ 200 cells/µL. This view is supported by Gupta et al.^17^ and the South African guidelines for chronic renal dialysis.^1^ No significant difference in bacteraemia rate, organisms implicated or outcome was noted between the HIV-infected and non-infected groups apart from the fact that HIV-infected patients took longer to recover from sepsis.
- The clinical and laboratory markers present in more than 50.0% of patients with line infection were raised white cell count, raised neutrophils, rigors, fever, tachycardia and raised C-reactive protein. Erythrocyte sedimentation rate is an unreliable marker, especially in HIV-infected patients where elevation is indicative of an ongoing inflammatory response. Acidosis is also uncommon and cannot be relied upon to detect sepsis in these patients.
- Similar antibiotic protocols for the treatment of CVC-RB in both HIV-infected and non-infected patients are encouraged. This study did not reveal major differences between the two groups with regard to the aetiology, the lag time to the onset of bacteraemia and the outcome, although HIV-infected patients took longer to clear their sepsis.
- There should be a low threshold for the removal of infected catheters. This policy has resulted in similar low morbidity and mortality in our population when compared to statistics from other parts of the world.
- Protocols that facilitate timely initiation of an elective, safe, optimal and permanent vascular access on patients with CKD stage four must be implemented to minimise the need for emergency dialysis, which carries a higher risk for bacteraemia by using temporary vascular access.
